# Casual effects of telomere length on sarcoidosis: a bidirectional Mendelian randomization analysis

**DOI:** 10.3389/fmed.2024.1408980

**Published:** 2024-07-17

**Authors:** Shiben Zhu, Ziyu Hao, Qihang Chen, Xiaoliu Liu, Wenyan Wu, Yanping Luo, Fang Zhang

**Affiliations:** ^1^School of Nursing and Health Studies, Hong Kong Metropolitan University, Kowloon, Hong Kong SAR, China; ^2^Jockey Club School of Public Health and Primary Care, The Chinese University of Hong Kong, Shatin, Hong Kong SAR, China; ^3^Medical Laboratory of Shenzhen Luohu People’s Hospital, Shenzhen, Guangdong, China; ^4^Department of Science and Education, Shenzhen Baoan Women's and Children's Hospital, Shenzhen, Guangdong, China

**Keywords:** Mendelian randomization, telomere length, sarcoidosis, casual association, two-sample analysis

## Abstract

**Background:**

Telomere length, crucial for genomic stability, have been implicated in various inflamm-aging diseases, but their role in sarcoidosis remains unexplored.

**Objective:**

This study aims to explore the casual effects between TL and sarcoidosis via a bidirectional Mendelian Randomization (MR) study.

**Methods:**

We examined single nucleotide polymorphisms (SNPs) associated with TL and sarcoidosis, utilizing available open-access genome-wide association study (GWAS) databases from the UK Biobank and FinnGen. We employed five MR techniques, including Inverse Variance Weighted (IVW), MR Egger, weighted median (WM), Robust adjusted profile score (RAPS), and Maximum likelihood, to assess causal relationships and explore pleiotropy.

**Results:**

Summary data extracted from GWAS datasets of TL (*n* = 472,174) and (*n* = 217,758) of European ancestry. Employing 130 SNPs with genome-wide significance as instrumental factors for TL, we detect a significant negative correlation between TL and sarcoidosis (OR: 0.682, 95% confidence interval: 0.524–0.888, p: 0.0045). Similarly, utilizing 6 SNPs with genome-wide significance as instrumental factors for sarcoidosis, we fail to identify a noteworthy association between sarcoidosis and TL (OR: 0.992, 95% confidence interval: 0.979–1.005, p : 0.2424).

**Conclusion:**

Our results suggest that longer telomeres may reduce the risk of sarcoidosis, highlighting TL as a potential biomarker for diagnosis and long-term monitoring. Understanding the critical role of telomere shortening enables more effective focus on diagnosing, treating, and curing sarcoidosis linked to telomeres. Clinical investigations into treatments that enhance TL are warranted.

## 1 Introduction

Sarcoidosis is a rare inflammatory disorder that affects different systems (1), leading to the development of granulomas and symptoms such as respiratory distress (2), dermatological issues (3), cardiac manifestations (4), and ocular complications (5). Without treatment, chronic and severe sarcoidosis affecting the heart, brain, or lungs can result in serious health consequences (6), significantly impacting quality of life and potentially leading to death (7). The prevalence ranges from 152 to 215 cases per 100,000 individuals, with incidence rates peaking in males aged 30 to 50 years and females aged 50 to 60 years (8). Most sarcoidosis patients experience spontaneous disease remission (9), but 20–50% suffer from persistent illness requiring long-term therapy (10). The incidence and severity of sarcoidosis may be underestimated due to mild symptoms or misattribution to other conditions.

Telomeres are crucial DNA-protein complexes located at chromosome ends (11) that protect genomic stability by maintaining repetitive “TTAGGG” sequences. They shorten with each cell division, leading to replicative senescence, genetic instability, and apoptosis when critically short (12). Consequently, TL can vary across different tissues (13). Peripheral leukocyte TL measurements from the UK Biobank’s 472,174 participants correlate well with TL in other tissues, including those affected by sarcoidosis (14, 15). Blood TL is considered a reliable proxy for TL in these tissues (16, 17). Previous research has explored the association between TL and various diseases and behaviours, such as cardiovascular diseases (18), type 2 diabetes (19), cancers (20), Alzheimer’s disease (21), chronic kidney disease (22), chronic obstructive pulmonary disease (23), and alcohol consumption (24). Telomere shortening, linked to chronic inflammation, is observed in autoimmune diseases like rheumatoid arthritis (25), ankylosing spondylitis (25), and systemic lupus erythematosus (26) and may serve as a biomarker for disease activity and a new treatment target. However, few studies have examined the relationship between TL and sarcoidosis.

Mendelian Randomization (MR) is an emerging epidemiological tool that provides valuable insights into causal relationships between exposures, biomarkers, or risk factors and outcomes, especially when conducting randomized controlled trials is not feasible or ethical (27). MR effectively mitigates residual confounding and reverse causation, offering more robust results compared to many other methods for analysing observational data (28). Previous studies using MR have yielded mixed results regarding the association between TL and various health outcomes. For instance, Wium-Andersen et al. (29) found that short telomeres were not associated with depression in prospective or causal genetic analyses. In contrast, Rode et al. (30) reported that short telomeres in peripheral blood leukocytes were linked to higher mortality in association studies, while genetically determined short telomeres were associated with lower cancer mortality but not with all-cause mortality. Ye et al. (31) provided evidence of a genetic predisposition to shorter leukocyte TL and an increased risk of Graves’ disease. Additionally, Liao et al. (32) demonstrated that shorter telomeres might increase susceptibility to multiple sclerosis, highlighting the significance of TL in autoimmune diseases. Despite these findings, there have been no MR studies exploring the causal link between TL and sarcoidosis.

Our aim is to estimate the causal associations between TL and sarcoidosis by performing bidirectional two-sample MR analysis. Our findings, utilizing a robust and extensive genome-wide association study (GWAS) dataset, furnish convincing proof that genetically forecasted extended TL diminishes the likelihood of sarcoidosis, whereas sarcoidosis does not inherently affect TL. These outcomes not only bolster our comprehension of the interaction between TL and sarcoidosis but also underscore TL’s potential as a valuable biomarker for sarcoidosis. Our insights from the findings have the potential to reshape our methods for preventing and addressing sarcoidosis, presenting fresh avenues for therapeutic interventions and personalized care strategies.

## 2 Methods

### 2.1 Study design

**Figure 1:**
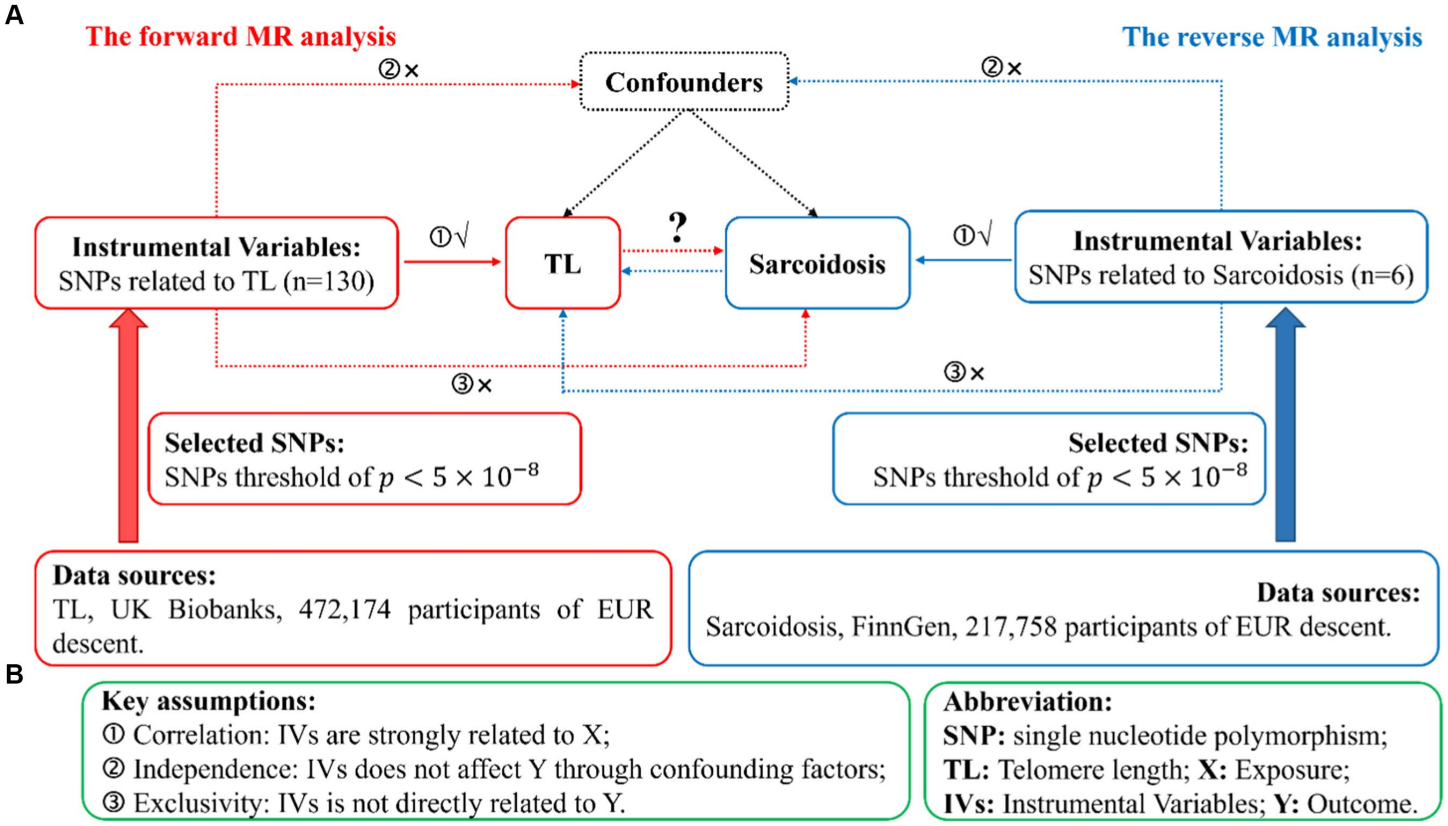
Illustration of study design in the bidirectional MR analysis. **(A)** Diagram outlining the study design. The red signifies the forward MR analysis, employing TL as the predictor and sarcoidosis as the consequence. The azure symbolizes the reverse MR analysis, utilizing sarcoidosis as the predictor and TL as the consequence. SNPs, single nucleotide polymorphisms; IVs, instrumental variables; TL, telomere length; MR, Mendelian randomization. **(B)** Three fundamental assumptions of MR analysis. X, exposure; Y, outcome.

We conduct a standard bidirectional MR analysis, utilizing SNPs significantly associated with the target as instrumental variables (IVs). Data extracted from GWAS datasets were utilized to evaluate the potential causal effects of the exposure on the outcomes. Given that genetic variations act as the foremost and efficient IVs in a MR analysis, qualified IVs must satisfy three basic assumptions outlined in MR theory. Figure 1A illustrates the comprehensive flowchart of this MR research, whereas Figure 1B portrays the fundamental MR suppositions and abbreviations. Additionally, the data for exposure and results were procured from distinct and autonomous samples.

### 2.2 Data source

We obtained all data from the IEU Open GWAS project, which is freely accessible to the public. After screening and the exclusion of redundant studies, and individuals of non-European ancestry, our analysis encompassed summary-level data from GWAS concerning genetic factors associated with TL, which prominently featured data from the UK Biobank (33). The information regarding genetic variants related to TL was extracted from a GWAS meta-analysis involving a cohort of 472,174 individuals, with a nearly equal distribution of males (45.8%) and females (54.2%), and European ancestry (34). Peripheral blood leukocytes were used to obtain DNA samples for TL measurement. The TL was measured using quantitative PCR (qPCR), as detailed in the studies by Codd et al. (15, 35). These studies ensured that the measurement process followed standardized protocols to maintain accuracy and reproducibility. Specifically, TL was assessed relative to a single-copy gene, with the telomere to single-copy gene (T/S) ratio used as the metric for TL. For sarcoidosis, SNPs were chosen as IVs from a GWAS dataset derived from the FinnGen project (36). The sample size consists of 217,758 individuals of European ancestry, including 2,046 cases and 215,712 controls. Table 1 furnishes a concise summary overview of the data in this study.

**Table 1:** An overview of the GWAS summary statistics.

Traits	Data source	Author & year	Sample size	Cases	Control	No. of SNPs	Sex	Ancestry	GWAS ID
TL	UK Biobank	Codd et al., 2021 (35)	472,174	0	472,174	20,134,421	Males and Females	European	ieu-b-4879
Sarcoidosis	FinnGen	NA, 2021	217,758	2,046	215,712	16,380,463	Males and Females	European	finn-b-D3_SARCOIDOSIS

### 2.3 Selection of IVs

The $p<5\times 10^{-8}$ threshold is a widely accepted standard in genetic epidemiology for declaring genome-wide significance (37, 38). Therefore, we used this threshold to identify SNPs significantly associated with both TL and sarcoidosis. Additionally, we applied stringent criteria to eliminate any linkage disequilibrium, utilizing a 10,000 kb aggregation window and setting the $r^{2}$ threshold at 0.001. Subsequently, we scrutinized each SNP for any potential deviations from fundamental assumptions ② and ③ by referencing the PhenoScanner database. To gauge the IVs strength, we computed the F -statistic for each SNP as well as for the entire set of SNPs. The F -statistic for a single SNP was calculated using the following formula (39) $F=\frac{b^{2}}{se^{2}}$ , with “ b ” representing the impact of the IV on the exposure, and “ se ” indicating the standard error of “ b .” Meanwhile, the total F -statistic followed this formula: $F=\frac{N-K-1}{K}\times \frac{R^{2}}{1-R^{2}}$ , where “ N ” stands for the sample size for the exposure, “ K ” represents the number of SNPs. The variable “ $R^{2}$ ” depicts the fraction of exposure variance elucidated by SNPs and is calculated using the formula $R^{2}=2eaf\left(1-eaf\right)b^{2}$ , where “ eaf ” represents the effect allele frequency of the SNP, and “ b ” signifies the SNP’s influence on the exposure. An F -statistic surpassing ten serves as a strong indicator of a substantial correlation between the SNP and the observed phenotype.

In addition, an important step in MR is to determine whether there is a SNP for the exposure that corresponds to the same gene and has an effect on the outcome. To prevent any distortions related to strand orientation or allele coding, we eliminated palindromic SNPs. Afterward, we harmonized exposure and outcome data, excluding palindromic SNPs with moderate allele frequencies. To address potential horizontal pleiotropy effects, we utilized MR Pleiotropy RESidual Sum and Outlier (MR-PRESSO) (40) and MR Egger (41). For each SNP, the MR-PRESSO outlier test computed a p -value to evaluate its significance in terms of pleiotropy, while the MR-PRESSO global test determined an overall p -value for horizontal pleiotropy. We sorted the SNPs in ascending order based on their MR-PRESSO outlier test *p*-values and successively removed them one by one. The MR-PRESSO global test was repeatedly conducted on the remaining SNPs each time an SNP was excluded from the list. This process continued until the p-value for the global test exceeded significance ( p>0.05 ). The final roster of SNPs that remained after the removal of pleiotropic SNPs was then employed in the subsequent MR analysis. Finally, we applied MR Steiger filters to exclude SNPs with an incorrect causal direction.

### 2.4 MR methods

In our study, we utilized five MR methods to ensure the robustness and accuracy of our results. The selection of these methods was strategic, aimed at addressing potential biases and validating our findings comprehensively. We primarily employed the Inverse Variance Weighted (IVW) random-effects model (42), which integrates Wald estimates from multiple genetic instruments, efficiently testing causal relationships under the assumption that all instruments are valid. To counter potential deviations from this assumption, we used the Weighted Median (WM) (43) estimator. This method provides a reliable estimate even if a significant portion of the data (up to 50%) is influenced by invalid instruments. We included MR Egger regression (41) to identify and adjust for pleiotropic effects, where genetic variants might impact the outcome via pathways not directly related to the exposure under study. We also applied the Robust Adjusted Profile Score (RAPS) (44) for its robustness against outliers and model misspecification, which enhances the reliability of our causal inferences. Lastly, we incorporated the Maximum Likelihood method (45) to optimize the use of statistical data, allowing for precise estimations by accommodating variations in instrument strength and heterogeneity among genetic instruments.

### 2.5 Statistical analysis

To examine the causal, connect between TL and sarcoidosis, we utilized the IVW random effects demonstrate as our essential approach. This strategy combines Wald gauges for each SNP in a meta-analysis system. Moreover, our expository techniques included the WM, MR Egger relapse, RAPS, and Most extreme probability methods to guarantee the strength of our discoveries, account for potential perplexing components and surreptitiously interferer, and assess the potential presence of pleiotropic effects.

We performed affectability investigations to affirm the unwavering quality of our MR discoveries. At first, we utilized Cochran’s Q test (46) to survey heterogeneity over SNPs, considering a *p*-value surpassing 0.05 as no heterogeneity. Moreover, we inspected the potential nearness of flat pleiotropy among IVs utilizing both the MR Egger caught and the MR-PRESSO Remaining Entirety of Squares (RSSobs). To guarantee the expository judgment of our think about, we connected the MR-PRESSO strategy to distinguish and redress for any potential exception SNPs. The limit for factual importance was set at a two-sided *p*-value of less than 0.05. In expansion, we conducted a leave-one-out investigation to guarantee that the MR comes about were not excessive impacted by any single SNP. The MR analyses were carried out using the TwoSampleMR, mr.raps, forestploter, and MR-PRESSO packages within the R statistical environment, version 4.3.2.

## 3 Results

### 3.1 Instrumental variables

In our forward MR study targeting TL as the exposure, we initially selected 154 SNPs as IVs. An examination using PhenoScanner revealed no associations of these SNPs with known confounders or outcomes. After excluding palindromic SNPs, a total of 130 SNPs remained viable for analysis. Neither the Steiger nor the MR-PRESSO outlier tests flagged any of these SNPs as aberrant. Each SNP in this refined group boasted an F-statistic exceeding ten, with the collective $R^{2}$ of these SNPs being 3.50%, and their aggregate F-statistic reaching 123.8934.

In the reverse MR analysis, with sarcoidosis as the primary focus, our initial choice encompassed 6 SNPs selected as IVs for sarcoidosis. Similar to the TL analysis, the PhenoScanner investigation indicated no association between these SNPs and potential confounding factors or outcomes. Post-removal of palindromic SNPs, all 6 SNPs persisted for analysis. The Steiger and MR-PRESSO corroborated the reliability of these SNPs, identifying none as outliers. Each of these SNPs had an F-statistic well above ten. The combined $R^{2}$ for these SNPs stood at 0.12%, with their total F-statistic being 41.90.

Supplementary Tables S1, S2 offer comprehensive insights into the initial selection of SNPs in PhenoScanner for our study. Meanwhile, Supplementary Table S3 compiles information on SNPs that did not meet the inclusion in the ultimate analysis. For a more comprehensive understanding, please refer to Supplementary Table S4, which presents exhaustive SNPs used in the final analysis. All the pertinent information can be found in the Supplementary materials.

### 3.2 MR results

**Figure 2:**
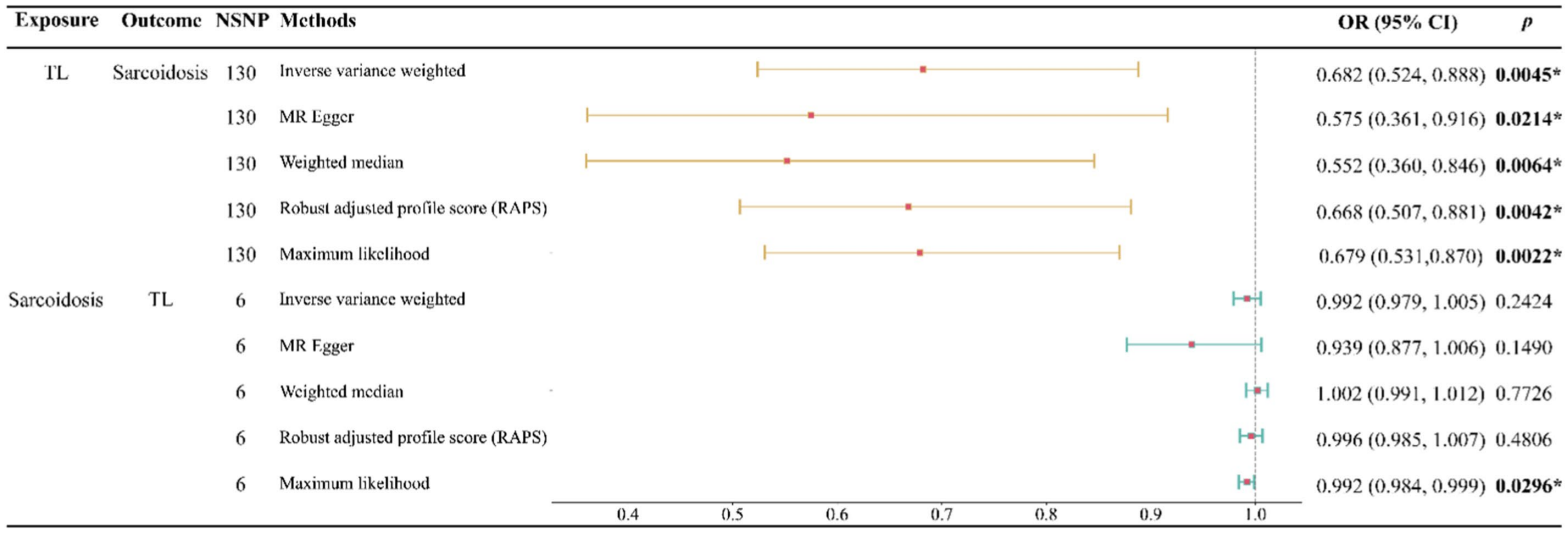
Forest plot of the bidirectional two-sample MR analysis. NSNP, number of SNPs; OR, odds ratio.

Our study indicates that a genetically predicted increase in TL may reduce the risk of developing sarcoidosis. This causal relationship is robustly supported across five analytical methods, including IVW, MR Egger, WM, RAPS, and Maximum likelihood, with each method consistently aligning in the direction of this causality (Figure 2). Specifically, when assessing TL as the exposure variable, the IVW analysis revealed a notably substantial adverse causal link with sarcoidosis (OR: 0.682, 95% confidence interval: 0.524–0.888, *p*: 0.0045), a finding echoed by MR Egger (OR: 0.575, 95% confidence interval: 0.361–0.916, *p*: 0.0214), Weighted Median (OR: 0.552, 95% confidence interval: 0.360–0.846, *p*: 0.0064), RAPS (OR: 0.668, 95% confidence interval: 0.507–0.881, *p*: 0.0042), and Maximum likelihood (OR: 0.679, 95% confidence interval: 0.531–0.870, *p*: 0.0022).

Conversely, when examining sarcoidosis as the exposure, our analyses found no significant evidence of its causal impact on TL. This non-causal relationship was consistent across multiple methods, including IVW (OR: 0.992, 95% confidence interval: 0.979–1.005, *p*: 0.2424), MR Egger (OR: 0.939, 95% confidence interval: 0.877–1.006, *p*: 0.1490), Weighted Median (OR: 1.002, 95% confidence interval: 0.991–1.012, *p*: 0.7726), RAPS (OR: 0.996, 95% confidence interval: 0.985–1.007, *p*: 0.4806), and Maximum likelihood (OR: 0.992, 95% confidence interval: 0.984–0.999, *p*: 0.0296) (Figure 2).

**Figure 3:**
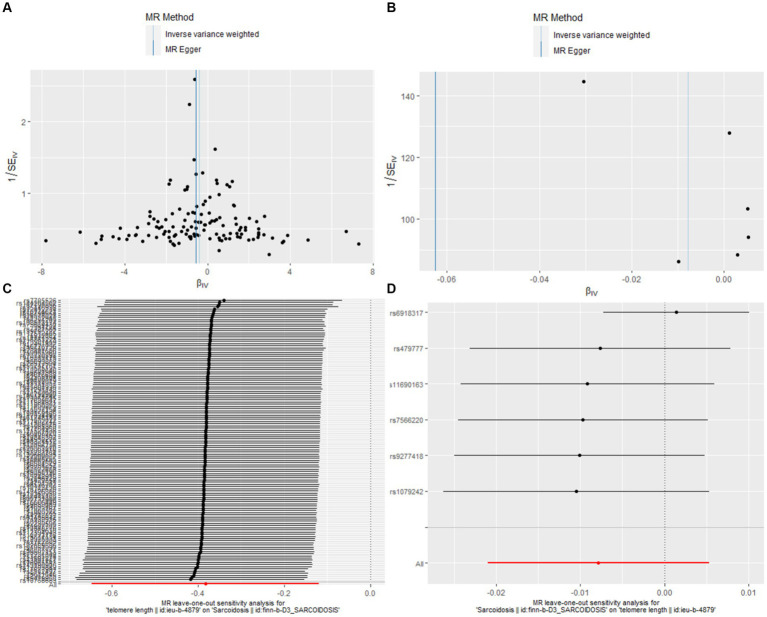
Leave-one-out and funnel plot visualizations between sarcoidosis and TL. **(A)** We utilized a funnel plot to assess the heterogeneity in the observed association during forward MR analysis. **(B)** A funnel plot was employed to examine the potential existence of significant heterogeneity in the observed association during reverse MR analysis. **(C)** Leave-one-out analyses were conducted to assess whether any IV exerted a substantial influence on the causal effect in forward MR. **(D)** Leave-one-out analyses were performed to determine whether any single IV played a pivotal role in driving the causal effect in reverse MR.

In our bidirectional MR analysis, both leave-one-out analyses and funnel plots enhance the nuanced interplay between TL and sarcoidosis (Figure 3).

Figures 3A,B, the funnel plots for forward and reverse MR respectively, were instrumental in assessing bias or heterogeneity within the observed associations. In the forward MR analysis (Figure 3A), the funnel plot displayed a symmetrical inverted funnel as cluster near the average effect size, evidenced by IVW analysis (b = −0.3829, se = 0.1346, *p* = 0.0045) and further supported by the MR Egger method (b = −0.5538, se = 0.2377, *p* = 0.0214). Conversely, a gap on one side of the funnel for the reverse MR analysis (Figure 3B) indicated smaller studies showing a particular kind of result are missing, with the IVW method showing (b = −0.0078, se = 0.0067, *p* = 0.2424) and the MR Egger method corroborating (b = −0.0626, se = 0.0351, *p* = 0.1490).

The Leave-one-out analyses, presented in Figures 3C,D, evaluated the robustness of these causal effects, ensuring that no single instrumental variable disproportionately influenced the outcomes. In the forward MR leave-one-out analysis (Figure 3C), the results mirrored the main analysis with a consistent negative association (b = −0.3829, se = 0.1346, *p* = 0.0045). Similarly, the reverse MR leave-one-out analysis (Figure 3D) provided further support for the absence of a causal relationship between sarcoidosis and TL (b = −0.0078, se = 0.0067, *p* = 0.2424).

**Figure 4:**
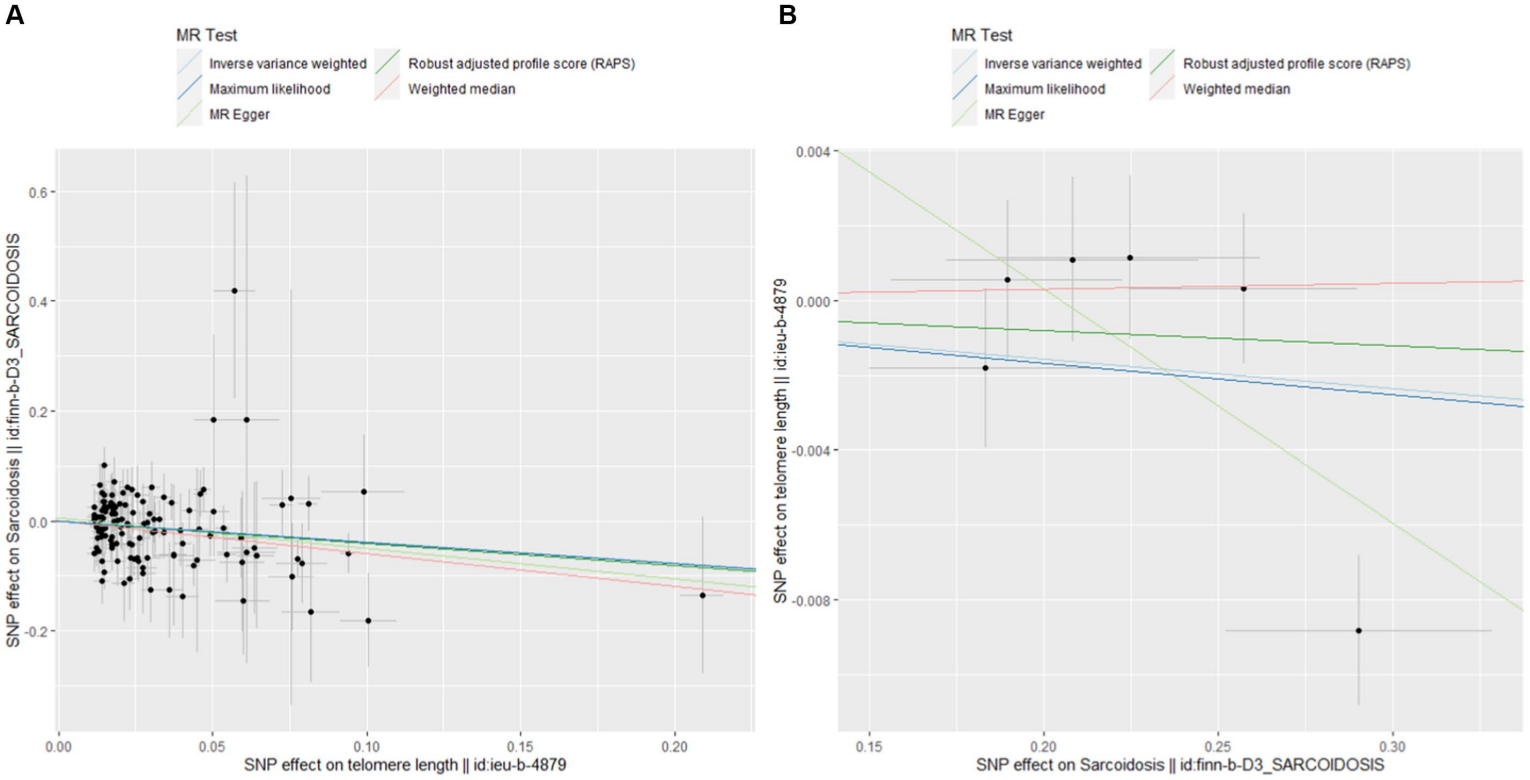
Scatterplots of MR analysis. **(A)** Scatter plot for forward MR analysis. **(B)** Scatter plot for reverse MR analysis.

Figure 4 shows pivotal scatter plots that easily elucidate the results of our MR analysis. Figure 4A visually depicts a downward trajectory between TL’s IVs and their impact on sarcoidosis, with the arrangement of points distinctly confirming a notable negative causal relationship. Conversely, Figure 4B illustrates no significant relationship, which focuses on the IVs for sarcoidosis and their impact on TL. The contrast between these two scatter plots in Figure 4 significantly reinforces our findings on the causal association between TL and sarcoidosis, highlighting the robustness and specificity of our bidirectional MR approach.

### 3.3 Sensitivity analysis

We examined heterogeneity and horizontal pleiotropy within our MR framework, with a comprehensive breakdown provided in Table 2. When analysing TL as the exposure on sarcoidosis, we utilized 130 SNPs, explaining 3.50% of the variance ( R2 ) with a robust F-statistic of 123.8934. Heterogeneity tests, including MR Egger and IVW, yielded Q values of 147.3561 (*p* = 0.1161) and 148.2342 (*p* = 0.1183), respectively, suggesting no significant heterogeneity. Pleiotropy evaluations, using MR Egger and MR-PRESSO, indicated negligible pleiotropic effects, with intercepts at 0.0060 (*p* = 0.3841) and an RSSobs of 150 (*p* = 0.1120), suggesting the absence of significant pleiotropic effects. Conversely, when we conducted the reverse MR analysis with sarcoidosis as the exposure variable and involving 6 SNPs, we observed the R2 value of 0.0012 and a substantial F-statistic of 41.90. This direction showed some level of heterogeneity, as evidenced by Q values of 9.9745 (*p* = 0.0409) for MR Egger and 16.2213 (*p* = 0.00624) for IVW, and suggested potential pleiotropic effects, with an MR Egger intercept of 0.0128 (*p* = 0.1886) and RSSobs of 29 (*p* = 0.0210).

**Table 2:** Heterogeneity and pleiotropy in our bidirectional MR analysis.

Exposure	Outcome	No. of SNPs	*R* ^2^	F-statistic	Heterogeneity				Pleiotropy			
					MR Egger		IVW		MR Egger		MR-PRESSO	
					Q	*p*-value	Q	*p*-value	Intercept	*p*-value	RSSobs	*p*-value
TL	Sarcoidosis	130	0.0350	123.8934	147.3561	0.1161	148.2342	0.1183	0.0060	0.3841	150	0.1120
Sarcoidosis	TL	6	0.0012	41.90	9.9745	0.0409	16.2213	0.00624	0.0128	0.1886	29	0.0210

## 4 Discussion

Using bidirectional MR, we demonstrated that decreased TL is associated with an increased risk of sarcoidosis, but there is no evidence of a reverse association in European-ancestry populations. Our findings remained consistent even when models accounting for violations of MR assumptions, such as confounding by pleiotropy, were applied. Recent studies underscore the importance of TL and biological age in various interstitial lung diseases, including pulmonary fibrosis (47–49) and rheumatoid arthritis-associated interstitial lung disease (ILD) (50). These studies indicate that shorter telomeres are linked to poorer treatment responses and worse clinical outcomes. Additionally, distinct gene expression signatures related to aging and senescence have been identified in conditions like scleroderma-associated ILD (51), suggesting potential therapeutic targets and the need for personalized treatment approaches.

Building on this, our comprehensive study analyzed large cohorts and found a negative association between TL and sarcoidosis across 130 genetic predictors of TL. We observed minimal evidence of heterogeneity and pleiotropy between variants in our cohorts. In instances where heterogeneity in the sarcoidosis cohort was noted, it was not driven by any single outlier SNP and showed no differing association with disease between the United Kingdom and Finland cohorts. These findings provide robust evidence suggesting a causal link between short telomeres and sarcoidosis, while also indicating divergent underlying disease mechanisms in sarcoidosis. Although sarcoidosis is not typically associated with families affected by telomere biology disorders (52), there are indications that short telomeres may lower the threshold for damage induced by chronic cigarette smoke (53), obesity (54), and alcohol (55). Therefore, while short telomeres may not have a direct causal role in sarcoidosis, they could contribute to the age-related onset of chronic immune-inflammatory responses.

Sarcoidosis is not typically considered an age-related condition, as it most commonly affects individuals between the ages of 25 and 45 (56). The prevalence of sarcoidosis does not significantly vary by sex or age, although it is more frequent in adults under 50, particularly among African American and Scandinavian populations (57). Interestingly, our findings indicate that while sarcoidosis does not impact TL (OR = 0.992, p =0.2424), longer TL significantly reduces the risk of developing sarcoidosis by 32% ( p =0.0045). Both interstitial lung disease and pulmonary fibrosis associated with sarcoidosis are an example of age-related diseases, with increasing attention on the role of accelerated aging and cellular senescence (58). Cellular senescence is a complex process that can be broadly categorized into replicative senescence, caused by intrinsic cellular events like telomere shortening, and stress-induced senescence, driven by factors such as oxidative stress and DNA damage (59, 60). Recent studies suggest that pulmonary fibrosis is associated with significantly shorter telomeres (61, 62). Shortened telomeres have also been found directly in lung tissue of patients with ILDs (63). Although there are mixed reports on the correlation between leukocyte TL and tissue TL (64–66), the study identified a significant positive correlation between leukocyte TL and lung biopsy TL in pulmonary fibrosis (67). Given our findings, we hypothesize that telomere shortening could act as an intrinsic and systemic driver of cellular senescence in sarcoidosis, similar to the association observed between telomere-associated driver mutations and pulmonary fibrosis.

Our study investigates the genetic link between TL and sarcoidosis, revealing that TL-related mechanisms may increase susceptibility to the disease. We found that shorter telomeres are associated with a higher risk of sarcoidosis, suggesting that TL could serve as a valuable biomarker for diagnosing or monitoring the condition. Conversely, longer telomeres appear to offer protection against sarcoidosis, indicating that individuals with genetically longer telomeres may have cellular environments less prone to inflammation. Recent study suggests shorter telomeres are linked to poorer outcomes and increased side effects of immunosuppression in ILDs, as shown by Mackintosh et al. (33). These findings imply TL might also influence treatment responses in sarcoidosis. Our results showing a negative correlation between TL and sarcoidosis risk support TL as a valuable biomarker for disease risk and treatment response, highlighting the need for further research in this area.

Similar to randomized controlled trials, MR leverages the principles of gene randomization and independent gene assortment to establish causal relationships between risk factors and health outcomes. With the burgeoning availability of GWAS summary statistics, MR has become instrumental in uncovering causal relationships between traits and diseases, thereby informing hypothesis-driven studies and clinical trial designs. This methodology is increasingly recognized for its robustness in the realm of epidemiological research. Our insights, extracted from comprehensive GWAS data, highlight the pivotal role of MR in unravelling the intricate genetic interplays that often elude detection through conventional observational studies. Thus, this approach not only enhances the foundational knowledge in genetic epidemiology but also sets forth a nuanced understanding of the genetic determinants underlying sarcoidosis, potentially guiding future clinical interventions and research trajectories in this domain.

Our research suggests a possible causal relationship between shorter telomeres and sarcoidosis, indicating new opportunities for therapeutic intervention. Strategies to improve telomere maintenance, either broadly or in specific cells critical to disease progression, could offer substantial benefits. However, restoring TL is complex and cannot be easily achieved by simply increasing telomerase levels due to increasing cancer risks. This remains true even with ongoing clinical trials in other aspects (68). To mitigate these risks, researchers are exploring safer methods to stimulate telomerase. One approach is the temporary administration of modified Telomerase reverse transcriptase (TERT) ribonucleic acid in cardiology, which prevents prolonged telomerase activity (69). While targeted delivery for pulmonary fibrosis remains challenging, ongoing advancements are expected to make this feasible in the future. Efforts to reduce the burden of senescent cells in sarcoidosis are also progressing. Senolytic drugs, such as masitinib combined with quercetin, are currently being studied in an open-label phase 1 trial (70). Additionally, other treatments, such as androgen therapy, show potential. Historically, testosterone has been effective in treating telomere-related diseases like aplastic anaemia. Danazol has shown promise in treating pulmonary fibrosis by halting lung function decline over a 36-month period (71). Furthermore, androgens can restore telomerase levels in cells from patients with TERT gene mutations associated with telomere disorders (72). Identifying TL as a biomarker for sarcoidosis risk could revolutionize early diagnosis and intervention strategies, potentially enabling personalized prevention and treatment approaches for individuals with genetically shorter telomeres.

However, our work has several limitations. The primary focus of our research is on individuals of European descent, which may limit its ability to capture the intricacies observed in other ethnic groups that are distinguished by distinctive cultural heritages and lifestyles. Furthermore, our investigation of TL is founded on GWAS data that was collected from peripheral blood leukocytes. It is imperative to emphasise that our data does not compare TL between fibrotic and non-fibrotic sarcoidosis, nor does it indicate whether telomere length was measured while individuals were undergoing therapy. This method may not accurately represent the variations in TL across distinct cell types or tissue subgroups that are significant to sarcoidosis. Additionally, the MR may have been biased due to the discrepancies in the sample sizes of the two primary databases that we employed. MR assesses causal concepts by randomly designating genetic variations. However, the potential impact of multiple polymorphisms on a variety of phenotypes presents a challenge in distinguishing between pleiotropy and mediation.

## 5 Conclusion

Our results suggest that longer telomeres may reduce the risk of sarcoidosis, highlighting TL as a potential biomarker for diagnosis and long-term monitoring. Understanding the critical role of telomere shortening enables more effective focus on diagnosing, treating, and curing sarcoidosis linked to telomeres. Clinical investigations into treatments that enhance TL are warranted.

## Data availability statement

Publicly available datasets were analyzed in this study. This data can be found at: https://gwas.mrcieu.ac.uk/.

## Ethics statement

Ethical approval was not required for the studies involving humans because the analyses conducted in this study exclusively relied on publicly accessible summary data, obviating the need for institutional review board approval. The studies were conducted in accordance with the local legislation and institutional requirements. The human samples used in this study were acquired from a database of genetic associations from GWAS summary datasets, for querying or download https://gwas.mrcieu.ac.uk/. Written informed consent to participate in this study was not required from the participants or the participants’ legal guardians/next of kin in accordance with the national legislation and the institutional requirements.

## Author contributions

SZ: Conceptualization, Data curation, Formal analysis, Methodology, Software, Writing – original draft, Writing – review & editing. ZH: Data curation, Formal analysis, Methodology, Writing – original draft. QC: Data curation, Methodology, Writing – original draft. XL: Data curation, Formal analysis, Methodology, Writing – original draft. WW: Data curation, Formal analysis, Writing – original draft. YL: Methodology, Writing – original draft. FZ: Conceptualization, Methodology, Project administration, Supervision, Writing – original draft, Writing – review & editing.
